# Fabrication of Laminated Micro/Nano Filter and Its Application for Inhalable PM Removal

**DOI:** 10.3390/polym15061459

**Published:** 2023-03-15

**Authors:** Wenhua Ma, Huan Qi, Yongmeng Zhang, Minggang Lin, Yiping Qiu, Chuyang Zhang

**Affiliations:** 1 College of Textile and Apparel, Xinjiang University, Urumqi 830049, China; 2 Institute of Smart & Ecological Textile, Quanzhou Normal University, Quanzhou 362002, China; 3 Key Laboratory of Clothing Materials of Universities in Fujian, Quanzhou Normal University, Quanzhou 362002, China; 4 College of Textile and Apparel, Quanzhou Normal University, Quanzhou 362002, China

**Keywords:** air filtration, nonwoven composites, quality factor, gradient layer filtration, electrostatic decay

## Abstract

Particulate matter (PM) with a diameter of 0.3 µm is inhalable and brings great threats to human health. Traditional meltblown nonwovens used for air filtration need to be treated by high voltage corona charging, which has the problem of electrostatic dissipation and thus reduces the filtration efficiency. In this work, a kind of composite air-filter with high efficiency and low resistance was fabricated by alternating lamination of ultrathin electronspun nano-layer and melt-blown layer without corona charging treatment. The effects of fiber diameter, pore size, porosity, layer number, and weight on filtration performance were investigated. Meanwhile, the surface hydrophobicity, loading capacity, and storage stability of the composite filter were studied. The results indicate that the filters (18.5 gsm) laminated by 10 layers fiber-webs present excellent filtration efficiency (97.94%), low pressure drop (53.2 Pa), high quality factor (QF 0.073 Pa^−1^), and high dust holding capacity (9.72 g/m^2^) for NaCl aerosol particles. Increasing the layers and reducing individual layer weight can significantly improve filtration efficiency and reduce pressure drop of the filter. The filtration efficiency decayed slightly from 97.94% to 96.48% after 80 days storage. The alternate arrangement of ultra-thin nano and melt-blown layers constructed a layer-by-layer interception and collaborative filtering effect in the composite filter, realizing the high filtration efficiency and low resistance without high voltage corona charging. These results provided new insights for the application of nonwoven fabrics in air filtration.

## 1. Introduction

Particulate matter (PM) is one of the most important sources of air pollution [1]. Tiny airborne particles generally exist in the form of solid dust, liquid droplets, aerogels, etc., and their chemical composition is extremely complex [2]. Dust particles with 0.3 μm diameter are the most penetrating particles, which are difficult to be captured by filter [3,4]. They can penetrate deeply into human’s lungs and bronchi, thus triggering various respiratory diseases such as asthma, pneumoconiosis, lung cancer and so on [5,6,7]. COVID-19 coronavirus is also airborne in the form of aerogel particles. As a result, people realize the necessity of wearing masks, especially in times of foggy weather and respiratory epidemics.

Masks are usually composed of nonwovens produced through spunbond, needle-punch, hot air bonding, electrospinning, and meltblown technologies. Among them, spunbond, needle-punch, and hot air bonding nonwovens are usually used as cover and supporting layer. While electrospinning and meltblown nonwovens generally serve as the core functioning layer due to small fiber diameter. Meltblown nonwovens usually require corona charging treatment to improve their filtration efficiency before use [8,9,10]. However, the electret charge decays easily in actual service environment especially with high temperature and humidity, which leads to a sharp decline in filtration efficiency [11,12]. This is why masks with electret meltblown nonwovens need to be replaced after 4 h of wearing. Various electrets were added to polymers to slow charge decay such as barium titanate [13,14], nanoboehmite [15], and magnesium stearate [16]. The incorporation of electrets enhances the stability of charge storage in meltblown, but to a certain extent it affects the spinnability of the polymer. Besides, electrets do not fundamentally solve the problem of charge attenuation.

Electrospun membranes have been investigated intensively in recent years [17,18,19]. However, there are still many drawbacks with pure electrospun membrane for air filtration, such as high cost, low dust holding capacity [20,21]. To overcome these drawbacks, a lot of research has been done such as surface structure design [22], fiber and pore size optimization [23,24] and fiber surface modification [25]. PVDF (Polyvinylidene fluoride) nano fibers were widely studied for energy harvesters and tactile sensors due to its piezoelectric property [26,27,28,29]. Moreover, it has high mechanical strength, high hydrophobicity, good flexibility, good chemical and thermal stability, and excellent aging resistance, which are important for its application in air filtration [30,31,32,33]. In existing studies, nanofiber membranes usually need to be stacked to a certain thickness to achieve high filtration efficiency, but the rise in membrane thickness can cause a sharp increase in filtration resistance, which limits their filtration performance. To the best of our knowledge, there are few reports on the lamination of melt-blown and nanowebs to fabricate micro/nano composite filter. The filtration performance of the composite filter needs to be investigated.

In this work, we report a novel method combining electrospinning and non-electret meltblown for preparing micro/nano filters to remove PM (0.3). PP microfibers and PVDF nanofibers were selected as building blocks due to their excellent comprehensive properties. The ultrathin meltblown layers and ultrathin nanofiber layers were alternately stacked to achieve high efficiency and low resistance. The percentage of nanofibers and microfibers was kept the same by regulating the spinning time and number of layers. The surface morphology was characterized on a scanning electron microscope (SEM). Pore size distribution was measured by a capillary flow porometer. Filtration performance was evaluated by an automated filter tester. Storage stability was assessed by charge decay and filtration efficiency decay. The filtration mechanism of composite filter was proposed according the results. This work provides a versatile strategy for further design and development of air filters with excellent filtration performance.

## 2. Materials and Methods

### 2.1. Materials

Polyvinylidene fluoride (PVDF, Mw = 1,000,000) was purchased from Huachuang Chemical Co., Ltd., (Foshan, China). N, N-dimethylformamide (DMF) was supplied by Yien Chemical Technology Co., Ltd., (Shanghai, China). Polypropylene pellets (MFI 1500) were supplied by China-Base Petrochemical Co., (Ningbo, China). All chemicals were used without further purification.

### 2.2. Sample Preparation

#### 2.2.1. Preparation of Meltblown

**Figure 1:**
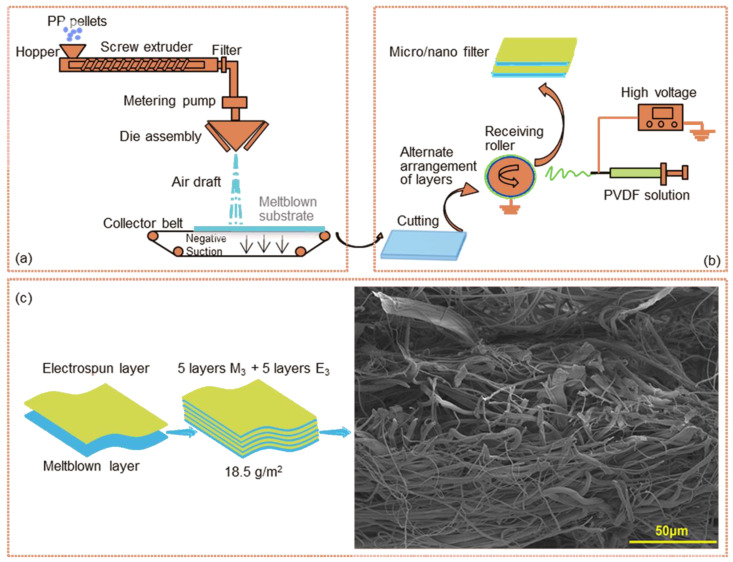
Fabrication of the composite filter (**a**) preparation of meltblown, (**b**) preparation of the laminated micro/nano filter, (**c**) structure diagram and cross-section SEM photography of M_3_E_3_*5.

Polypropylene (PP) meltblown was fabricated on the Spunbond-Meltblown-spunbond (SMS) pilot line in laboratory. Three kinds of meltblown (M_1_, M_2_, M_3_) with different base weights were fabricated by regulating the speed of collector belt to 10, 30, 50 m/min, respectively. Other parameters such as spinning temperature, drawing air temperature, air pressure, die to collector distance remained unchanged. The prepared meltblown were cut into dimensions of 31 × 20 cm to match the receiving roller on the electrospinning device. The spinning temperature and drawing air temperature were 260 °C and 270 °C, respectively. The pressure of drawing air was 0.1 MPa. The die to collector distance was 300 mm. The schematic process for meltblown is shown in Figure 1a.

#### 2.2.2. Preparation of Laminated Filter

PVDF powder was dissolved in DMF by continuously stirring for 10 h at room temperature. The 5 mL syringe loaded with PVDF solution (18 wt%) was placed on an injection pump for electrospinning. The meltblown was wrapped on a roller as the substrate for nanofibers. After receiving the nanofibers for a certain period of time, another piece of meltblown with the same base weight was laid on top to receive nanofibers. In this way, desired layer-by-layer micro/nano filters with homogeneous structure can be obtained. All fabricated filters were vacuum-dried at 80 °C for 10 h to remove residual solvent. The injection speed, collecting distance, and applied voltage were 1.0 mL/h, 15 cm, and 25 kV, respectively. The preparation process was carried out at the temperature of 25 ± 2 °C and relative humidity of 50 ± 5%. The preparation schematic of laminated micro/nano filters is shown in Figure 1b.

### 2.3. Structure Design

Three kinds of laminated filters were fabricated. All samples have the same base weight (18.5 g/m^2^) of microfibers (16.5 g/m^2^) and nanofibers (2.0 g/m^2^) and are marked as M_1_E_1_*1(1 layer of Microfiber/1 layer of nanofiber), M_2_E_2_*3(3 layers of Microfiber/3 layers of nanofiber), M_3_E_3_*5(5 layers of Microfiber/5 layers of nanofiber). The base weight of M_1_, M_2_, and M_3_ were 16.5 g/m^2^, 5.5 g/m^2^, and 3.3 g/m^2,^ respectively. For example, five layers of equivalent meltblown and five layers of equal nanofiber membrane are alternately laminated for composite filter M_3_E_3_*5. The cumulative electrospinning time for all micro/nano filters materials was 30 min (30 mins*1, 10 mins*3, 6 mins*5 for M_1_E_1_*1, M_2_E_2_*3, M_3_E_3_*5, respectively). Detailed parameters of these samples are listed in Table 1. The structure diagram and SEM image of M_3_E_3_*5 are shown in Figure 1c.

### 2.4. Characterization

The surface morphology of micro/nano filters was investigated by scanning electron microscope (TESAN MIRA LMS, Tescan China Ltd., Shanghai, China) after coating with gold. Fiber diameter distribution of the PVDF nonofibers and PP microfibers were analyzed on Nano Measure 1.2. The thickness of micro/nano filters was measured by fabric thickness gauge (YG141D, Quanzhou City Meibang Instrument CO., Ltd., Quanzhou, China). Five different positions were tested on each sample. Surface chemical elements were analyzed by energy dispersive x-ray (EDX) detector (Thermo Scientific Helios 5 CX, Thermo Fisher Scientific, Waltham, MA, USA). The chemical composition was recorded on Attenuated total reflectance infrared (ATR-IR) spectroscopy (Thermo Scientific Niolet iN10, Thermo Fisher Scientific, Waltham, MA, USA). Water contact angle (WCA) was recorded on CA-100A (Shanghai Innuo Precision Instruments Co., Ltd., Shanghai, China). The pore size distribution of filter was measured by a capillary flow porometer (CFP-1500-AEXL, Porous Materials Inc., Ithaca, NY, USA). The porosity of composite filter was tested by automatic true density analyzer (BSD-TD, Beishide Instrument Technology Co., Ltd., Beijing, China) through gas expansion method with helium. The surface potential of composite filter was tested on a non-contact electrostatic fieldmeter (FMX-004, Simco Japan Inc., Kobe, Japan). The distance between the filter and the probe was 2.5 cm. Twenty different positions were tested on each sample.

### 2.5. Filtration Performance

The filtration efficiency and pressure drop were measured by an automated filter tester (DR251XL, Wenzhou Darong Textile Instrument Co., Ltd., Wenzhou, China). Charge-neutralized sodium chloride (NaCl) aerosol particles were generated from 2.0 wt% NaCl aqueous. The particle sizes were normally distributed. The count median diameter of sodium chloride aerosol particles was 0.075 µm, and the geometric standard deviation of the particles was less than 1.86. They were dried after coming out from the Collision Nebulizer and then fed into the filter holder. Aerosol particles passed through the testing area of 100 cm^2^ at a flow rate of 32 L/min. The concentration of NaCl aerosols in the upstream and downstream of the filters was monitored by a photometer. The filtration efficiency (*η*) was calculated using the following equation:

(1) $$\eta =\frac{C_{up}-C_{down}}{C_{up}}\times 100\%$$

where *C_up_* and *C_down_* represent the concentration of NaCl aerosols in the upstream and downstream, respectively.

The quality factor (*QF*) is considered as a comprehensive parameter of filtration efficiency and pressure drop. The pressure drop of NaCl aerosols passing through the filter was continuously measured by the electronic pressure transmitter. *QF* can be calculated by the following equation:

(2) $$QF=-\frac{\mathrm{Ln}\left(1-\eta \right)}{\Delta P}$$

where Δ*P* represents the pressure drop. Three different flat and wrinkle-free areas were selected for the test. The average values were used as the final data to evaluate the filtration performance. 

Filtration loading test was performed to investigate the dynamic filtration property according to GB 2626-2019. To avoid extremely long testing time, the concentration of NaCl aerosol was adjusted up to 20 mg/m^3^ and the air flow rate was adjusted up to 85 L/min. The dust holding capacity has a great influence on the service life of an air filter, as it affects the filter replacement cycle directly. It can be measured through filtration loading test. The weight increase in an air filter when pressure drop reaches a specific value during loading test reflects its dust holding capacity. According to EN779-2002 standard, 450 Pa was selected as the limit in this work. In addition, storage stability was evaluated by testing the filtration efficiency every 20 days within an 80 day period.

## 3. Results and Discussion

### 3.1. Morphology of Laminated Micro/Nano Filter

**Figure 2:**
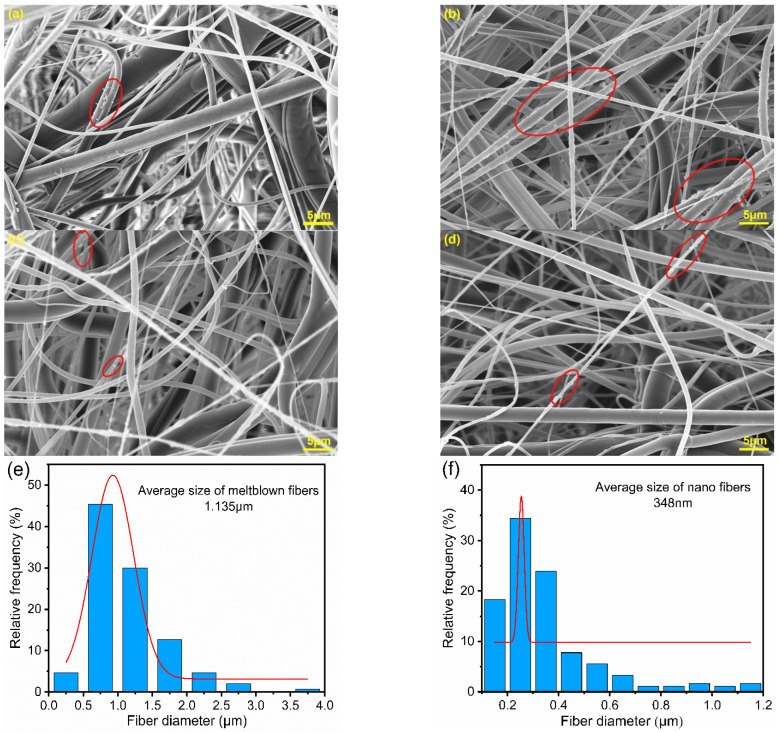
SEM images after filtration (**a**) bottom of pure meltblown M_1_, (**b**–**d**) filtrated bottom layers of M_1_E_1_*1, M_2_E_2_*3, M_3_E_3_*5, respectively. Fiber diameter distribution (**e**) meltblown microfibers, (**f**) electrospun nanofibers.

The bottom surface morphology of meltblown after filtration is shown in Figure 2a. Sodium chloride particles are rarely observed on meltblown fibers due to their poor interception capacity. While in Figure 2b, a large number of particles can be observed on the bottom nanofibers, indicating that the introduction of nanofibers significantly enhanced the interception capacity for particles. In Figure 2c, it can be found that there were apparently fewer particles on the bottom nanofibers of M_2_E_2_*3 compared to M_1_E_1_*1. This is due to the fact that the alternating arrangement of multilayer nanofibers and microfibers formed a gradient layer filtration effect. Most of the particles were already captured before they reached the bottom. As shown in Figure 2d, M_3_E_3_*5 had the least number of particles on the bottom layer, which further confirmed this inference. The apparent hierarchical structure consisting of random microfibers and nanofibers can be observed in M_1_E_1_*1 due to the alternating arrangement of different layers. However, in M_2_E_2_*3 and M_3_E_3_*5, the hierarchical structure gradually became less obvious and mixed with each layer. This blended structure will facilitate the synergistic effect of micron and nanofibers and improve the filtration performance.

As shown in Figure 2e, most of the fibers in meltblown were less than 1.5 μm in diameter. The average diameter was 1.135 μm and about 50% fibers were less than 1.0 μm. The reduction in fiber diameter in meltblown is beneficial to improve the ability to intercept particles and contribute more filtration efficiency for the filters. The fiber diameters of electrospun nanofibers were mostly smaller than 400 nm and the average fiber diameter was 348 nm (Figure 2f). The combination of meltblown and electrospinning technologies endows micro/nano filters with versatile fiber diameters, which is conducive to the formation of complex and variable pore structures and increases the tortuosity of the pores. In the multilayer structure, microfibers act as scaffolds and coarse filters while the nanofibers act as fine filters. It integrates the advantages of microfibers and nanofibers to achieve better synergy, which can improve the comprehensive performance of the composite.

### 3.2. Chemical Characterization

**Figure 3:**
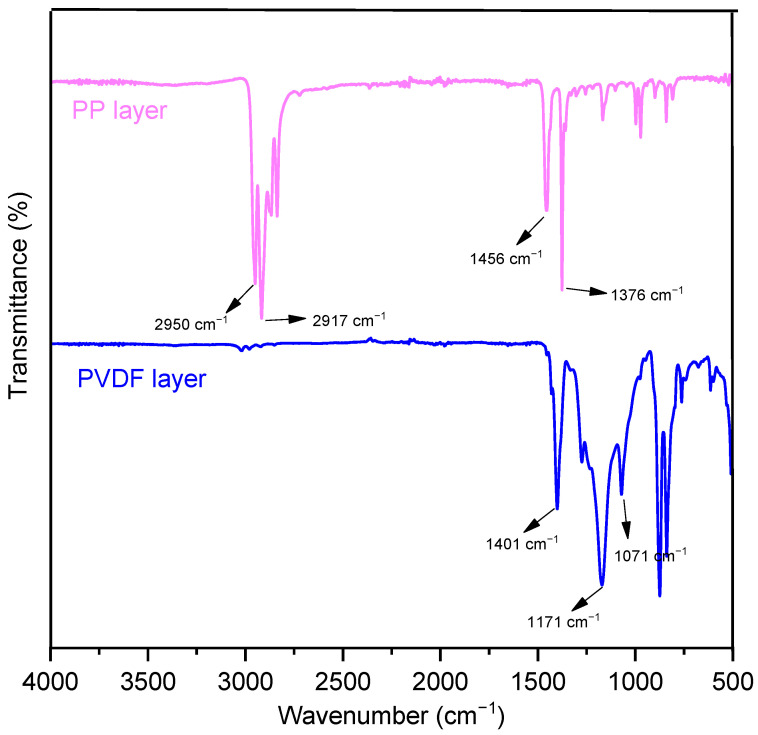
ATR-IR analysis of the composite filter.

The ATR-IR spectra of composite filter were recorded (Figure 3). The signals at 2950 cm^−1^ and 2917 cm^−1^ were the symmetric and asymmetric stretching vibration and 1456 cm^−1^ was the bending vibration of CH_2_, respectively [34]. The signal at 1376 cm^−1^ was the symmetric deformation vibration of CH_3_ in polypropylene [35]. The characteristic peak at 1401 cm^−1^ of polyvinylidene fluoride was associated with CH_2_ wagging mode [36]. The signals at 1171 cm^−1^ and 1071 cm^−1^ were assigned for the asymmetric and symmetric stretching vibration of CF_2_, respectively [37]. From the results, no new functional groups appeared in the composites after laminating. 

**Figure 4:**
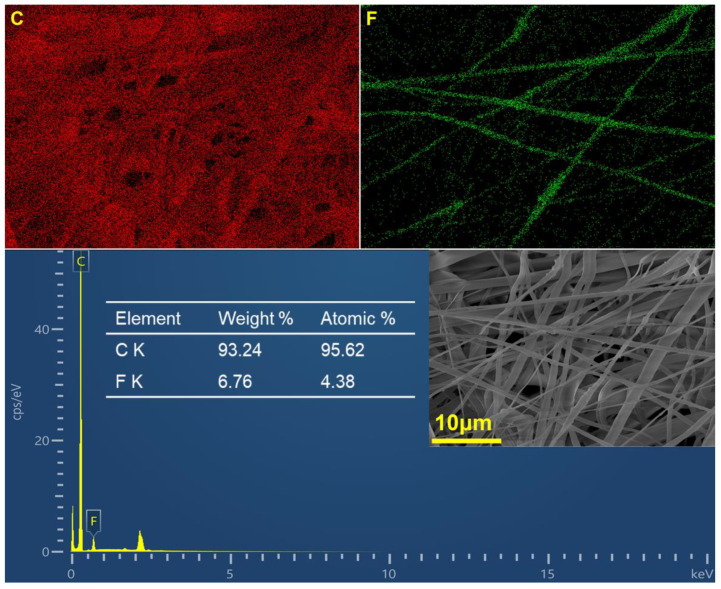
EDX images of the composite filter.

The surface elemental content of composite filter M_3_E_3_*5 was recorded on EDX spectra (Figure 4). The content (wt%) of Carbon element was 93.24%, which is due to the carbon backbones in PP and PVDF. The content of Fluorine element was 6.76%. That is because the content of PVDF nanofibers is 10.8% of the composites. The green phosphorescence bands in Fluorine mapping can be observed. This was due to the fact that the surface PVDF nanofibers were close to the probe and formed the clear dot bands, while others were partially covered and showed randomly distributed points. This inference can also be confirmed in the SEM image results. In general, the uniform distribution of elements in the EDX mapping confirmed the homogeneous structure of the filter, which is the key to the reliable filtration properties. 

### 3.3. Hydrophobicity

**Figure 5:**
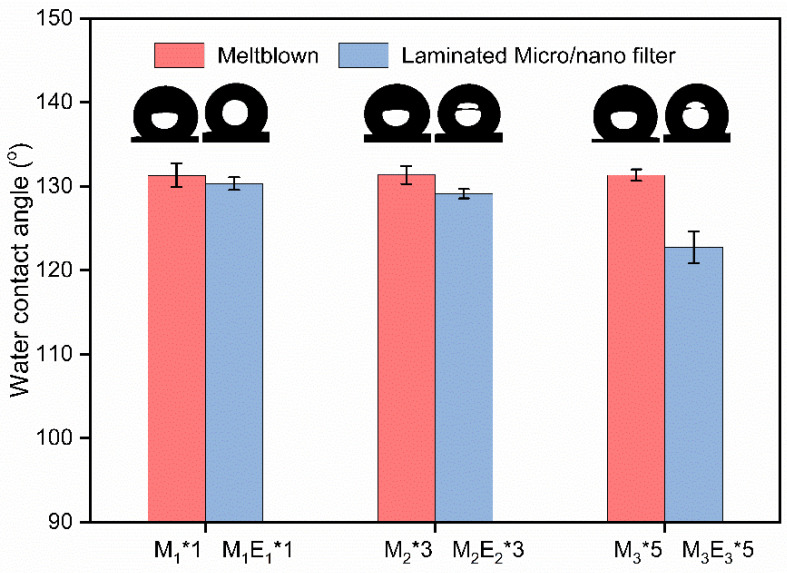
Water contact angles of samples.

Water contact angles of five different positions were measured on every sample and the results were shown in Figure 5. The bottom nanofilm of micro/nano filter was taken as the test area. From the results, the water contact angle of M_1_*1, M_2_*3, and M_3_*5 were almost equal (around 130°). That is because polypropylene is hydrophobic and these three samples have the same base weight and spinning parameters. The water contact angles of composite filters were slightly lower than those of meltblown. This result may be attributed to the high specific surface area of PVDF nanofibers, which increaed the surface energy and improved hydrophilicity [38,39]. The improvement of hydrophilicity will be beneficial for the spreading and transmission of water vapor. Besides, the contact angle of filters dropped slightly with the layers. This is due to the fact that the nanofiber layer contacted with water became sparse and thus reduced the support for water. The sparse nanofiber network was not favorable for the formation of a lotus leaf-like bionic structure, which weakened the synergistic hydrophobic effect.

### 3.4. Pore Size and Porosity

**Figure 6:**
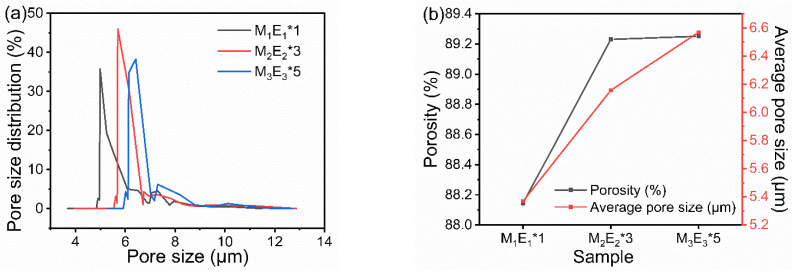
Composite filter (**a**) Pore size distribution and (**b**) porosity and average pore size.

The pore size distributions of micro/nano filters are shown in Figure 6a. The pore sizes of M_1_E_1_*1, M_2_E_2_*3, and M_3_E_3_*5 were mainly concentrated in 5–7 µm. The peak value increased from 4.98 µm to 6.42 µm, which reflected that the content of larger pores was increased. This is because the filter becomes fluffier with the increasing layers as verified in Figure 6b. The porosity increased from 88.14% to 89.25%. Meanwhile, the average pore size increased from 5.37 to 6.57 µm. The multilayer structure in filter facilitated the formation of contact interfaces, and thus improved the porosity and pore size. In general, micro/nano filters have high porosity and multi-scale pore sizes, which is essential to achieve high efficiency and low resistance.

### 3.5. Filtration Properties

**Figure 7:**
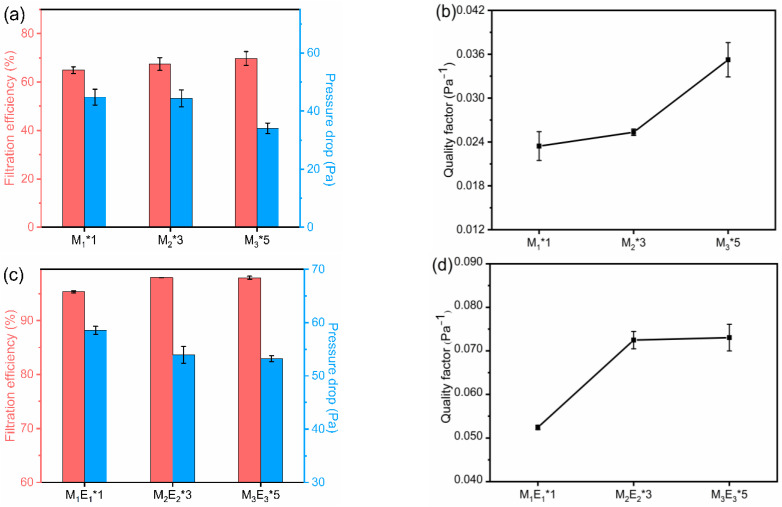

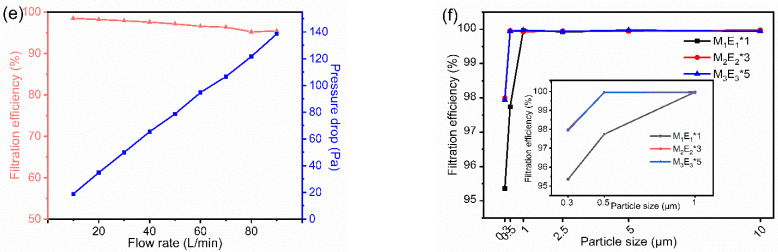
Meltblown filters (**a**) filtration efficiency, pressure drop, and (**b**) quality factor. Laminated micro/nano filters (**c**) filtration efficiency, pressure drop, and (**d**) quality factor. s. (**e**) Filtration efficiency and pressure drop of M_3_E_3_*5 with different flow rates. (**f**) Filtration efficiency versus the particle size. The detection particle size is 0.3 µm in (**a**,**c**,**e**).

As shown in Figure 7a,c, the filtration efficiencies of multilayered pure meltblown filters M_1_*1, M_2_*3, and M_3_*5 were 64.88%, 67.43%, and 69.79%, respectively. They tend to increase with the increasing number of layers, but they were all lower than 70%. That is because the large diameter results in large inter-fiber pores that allow particles to pass through easily. That is why meltblown usually needs corona charging treatment when used as an air filter. The filtration efficiencies of M_1_E_1_*1, M_2_E_2_*3, and M_3_E_3_*5 were 95.60%, 97.99%, and 97.94%, respectively. The introduction of nanofibers significantly improved the filtration efficiency. It is due to the small diameter of nanofibers, which can form smaller interfiber pores and facilitate the sieving and interception of particles. Secondly, the super high specific surface area of nanofibers can increase the probability of suspended particles to diffuse to their surface and deposit. Besides, the filtration efficiency showed a growing trend with increasing number of layers. The reason for this is that the multilayer structure increases the thickness of the composite filter (Table 1) and consequently increases the total length of the curved connecting pores, which facilitates the deposition of particles by collision. Inevitably, the pressure drop increased from 44.8, 44.4, 34.1 to 58.6, 53.9, 53.2 pa, respectively. This is attributed to the fact that the introduction of nanofibers increased the coverage and reduced the average pore size between fibers, which impedes the air flow. Comparing Figure 7b and Figure 7d, the quality factors (QFs) of micro/nano filters were significantly higher than that of meltblown filters. Moreover, the QFs of micro/nano filters rose to 0.073 Pa^−1^ when the number of layers increased to 10. This result is similar to those in the literature [40,41]. The layer-by-layer micro/nano structure enhanced the tortuosity of pores and endowed the sample with multi-scale pore sizes and diameters, thus improving the ability to capture particles. In addition, the larger pore size and porosity resulted in a lower pressure drop compared to M_1_E_1_*1 and M_2_E_2_*3. These two factors gave M_3_E_3_*5 a satisfactory quality factor. As shown in Figure 7f, micro/nano filters had lower filtration efficiencies for 0.3 μm NaCl aerosols. Their filtration efficiencies for particles larger than 0.3 μm almost all exceeded 99%. The filtration efficiency and pressure drop of M_3_E_3_*5 were tested at various flow rates in Figure 7e. The filtration efficiency decreased to 95.39% when the flow rate rose to 90 L/min. This is because the particles retention time in the filter was shortened for the high flow rate, which reduced the possibility of particles being captured [42]. Besides, the pressure drop increased almost linearly with the increase in flow rate, which was coincident with Darcy’s law [43].

### 3.6. Loading Performance and Storage Stability

**Figure 8:**
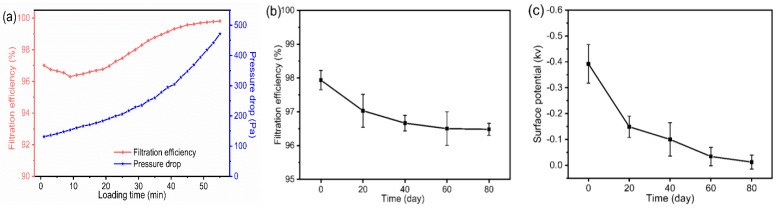
(**a**) Filtration efficiency and pressure drop during loading. Decaying test of M_3_E_3_*5 within 80 days, (**b**) Filtration efficiency decay, (**c**) Surface potential decay. The detection particle size is 0.3 µm in (**a**,**b**).

In Figure 8a, the filtration efficiency decreased from 97.02% to a minimum of 96.30% in the first 9 min and then gradually increased to almost 100%. This can be explained as follows: electrospinning had a certain electret effect, which made nanofibers contain a small amount of electrostatic charge and thus enhanced the ability to capture particles by static adsorption. However, during the loading test, the airflow carrying sodium chloride aerosols continuously rubbed against the micro/nano filter, causing the charge on the fibers to be transferred. In addition, the electrostatic adsorption was also weakened due to the shielding of fiber charge with increasing deposition of particles [44,45,46]. Despite this, the minimum value was still greater than 95%, which meets the standard of N95 masks. This stage was dominated by depth filtration and the pressure drop increased slowly. After that, it entered the transition stage and the surface cake filtration stage [47]. The mechanical interception was enhanced as captured particles became new sites for capturing more particles [48,49]. Meanwhile, the pressure drop showed a faster increase. That was because more and more pores were clogged with continuous deposition of particles, which obstructed airflow through. Bridges or branched chains formed by deposited particles during filter cake formation can perturb the flow, which was reported to be another possible mechanism leading to higher pressure drop [50,51]. It took 55 min for pressure drop to reach 450 Pa at a NaCl aerosol concentration of 20 mg/m^3^. When the concentration of PM 2.5 is higher than 0.25 mg/m^3^, it is defined as the most severe level of air pollution. The test concentration is 80 times on this value, so the actual use time will be significantly longer than the test time. At the same time, the dust holding capacity reached 9.72 g/m^2^, which is almost 53% of its base weight. The excellent filtration performance and robust dust holding capacity indicate a long service life. The filtration efficiency was measured every 20 days to evaluate its storage stability. As shown in Figure 8b, it decayed from 97.94% to 96.48% after 80 days, a reduction in only 1.49% of the initial value. The decay rate was relatively fast for the first 20 days and remained almost unchanged in the following 40 days. The results demonstrated the superior storage stability of laminated micro/nano filters. In Figure 8c, the surface potential of newly prepared sample was −0.392 kV, which was much lower than the reported electret filters [52,53]. It decayed fastest in the first 20 days and then decreased gradually, which was similar to the trend of filtration efficiency. After 80 days storage, the surface potential was −0.01 kV, which means that almost all of the charge generated during the electrospinning process escaped from the sample. However, M_3_E_3_*5 still had a high filtration efficiency of 96.48%. This is because the electrostatic adsorption of M_3_E_3_*5 is negligible compared to mechanical interception effects such as interception, inertial impaction, Brownian motion and gravitational settling [54]. The charge decay curve revealed the mechanism of filtration efficiency decay and further demonstrated the stable filtration performance.

### 3.7. Filtration Mechanism

**Figure 9:**
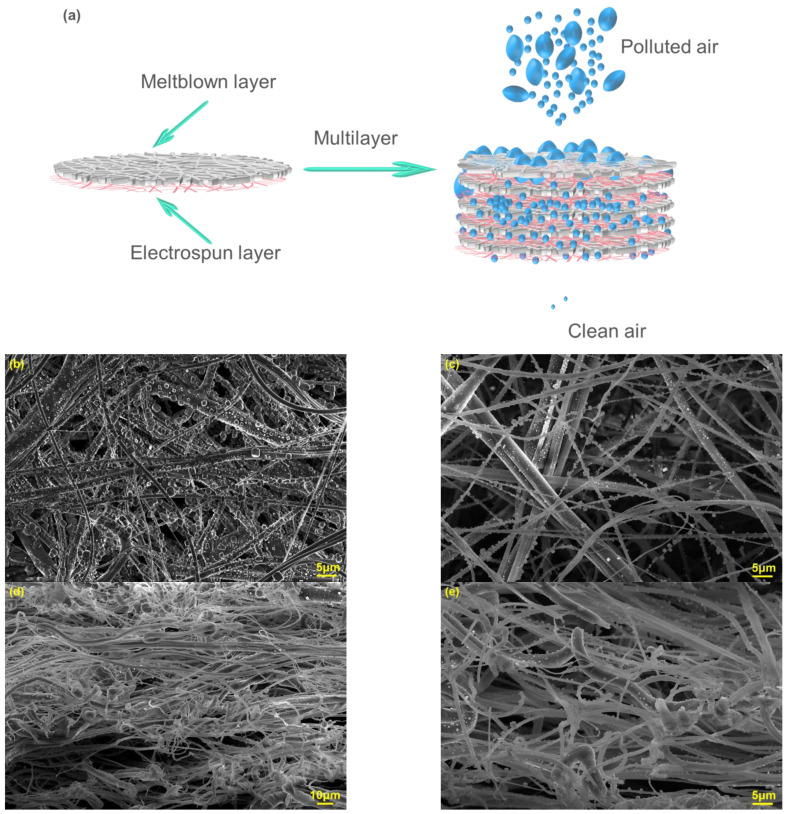
(**a**) Schematic diagram of filtration process. SEM images of M_3_E_3_*5 after loading (**b**) surface meltblown layer, (**c**) bottom nano layer, and (**d**,**e**) the cross section at different magnifications.

The filtration mechanism of the composite filter is illustrated in Figure 9a. The alternating lamination of micro/nano fiber-webs in ultrathin layers integrated the advantages of microfibers and nanofibers and enabled the materials to form a 3D structure, which enhanced their synergistic filtration effect. Thus, a gradient layer filtration can be achieved. In the filtration process, surface meltblown suffers the impact of airflow to maintain the stability of structure. It acts as a coarse filter to capture large particles as shown in Figure 9b. The presence of a coarse filter can significantly reduce the pressure drop growth rate of the fine filter and also effectively increase the total dust holding capacity of the whole filtration system and thus extend the service life [55]. Under the effect of inertia and gravity, most of the large particles are directly intercepted and deposited to the surface layer of the filter (Figure 9b). Since the size of fine particles is much smaller than filter pores, they mainly diffuse by Brownian motion from the surface layer to the bottom. However, a small amount of fine particles can be seen deposited on the nanofibers as shown in Figure 9c. This is due to the addition of multi-layer nanowebs in the composite, which can effectively capture the fine particles (~0.1 μm) by electrostatic adsorption and Brownian diffusion effect and improve the filtration efficiency [56]. The introduction of multilayer nanowebs also improves the layer-by-layer interception of the composite filter from the cross-section figures as shown in Figure 9d–e. The synergistic reaction mechanism of microfibers and nanofibers is the key to possessing excellent filtration performance.

## 4. Conclusions

In summary, the composite filters (18.5 gsm) were successfully fabricated by laminating 10 layers fiber-webs and exhibited satisfied filtration efficiency, low pressure drop, high quality factor, and dust holding capacity for NaCl aerosol particles. The ultrathin micro/nano fiber-webs in filters integrated the advantages of microfibers and nanofibers and enabled the composite to form a 3D structure with fine fibers, small pore size and high porosity. The increasing layers and thinning fiber-webs can improve the filtration efficiency and lower the pressure drop of the composite. The filtration efficiency of the composite filter still reached up to 96.48% after 80 days storage, avoiding the a decrease in the filtration efficiency due to charge decay on electret meltblown materials. The alternate arrangement of ultra-thin nano and melt-blown layers constructed a layer-by-layer interception and collaborative filtering effect in the composite filter, realizing the high filtration efficiency and low resistance without high voltage corona charging. The composite filters developed in this work are promising for application in the field of air filtration.

## Figures and Tables

**Table 1:** Parameters of laminated samples.

Sample	Number of Layers	Thickness (mm)	Base Weight (g/m^2^) (Microweb) (Nanoweb)		Total Base Weight (g/m^2^)
M_1_E_1_*1	2	0.158	16.50	2.00	18.50
M_2_E_2_*3	6	0.169	5.50*3	0.67*3	18.50
M_3_E_3_*5	10	0.192	3.30*5	0.40*5	18.50

## Data Availability

The data presented in this study are available on request from the corresponding author.
